# Supplementary material for: Elevated congenital heart disease birth prevalence rates found in Central Vietnam and dioxin TCDD residuals from the use of 2, 4, 5-T herbicides (Agent Orange) in the Da Nang region
Source: PLOS Glob Public Health. 2022 Oct 12;2(10):e0001050. doi: 10.1371/journal.pgph.0001050 (PMC10021360; doi:10.1371/journal.pgph.0001050)
Supplement: Attachment — Submitted filename: PLOS Response Letter.docx [file pgph.0001050.s001.docx]

Jose Ignacio Nazif-Munoz, Ph.D.
Academic Editor
PLOS Global Public Health

Re: **PGPH-D-22-00454
Elevated congenital heart disease birth prevalence rates found in Central Vietnam and dioxin TCDD residuals from the use of 2, 4, 5-T herbicides (Agent Orange) in the Da Nang region.
PLOS Global Public Health**

August 18, 2022

Dear Dr. Nazif-Munoz:

Thank you for the opportunity to revise and resubmit our manuscript to PLOS Global Public Health. We appreciate the reviewers’ careful reading and recommendations for improving the paper. Below are our responses to the issues raised by reviewers and revisions we have made. We think this is an important topic that needs to be published and discussed in a public forum and PLOS Global Health seems a perfect journal to start this discussion. Thanks for your consideration.

We address the journal requirements and reviewers’ comments as follows:

**Journal Requirements**

1. A marked-up copy of your manuscript that highlights changes made to the original version. You should upload this as a separate file labeled 'Revised Manuscript with Track Changes'. An unmarked version of your revised paper without tracked changes. You should upload this as a separate file labeled 'Manuscript'.

**Response:** Both of these have been uploaded in conjunction with this letter.

2. In the online submission form, you indicated that "All data will be available on request". All PLOS journals now require all data underlying the findings described in their manuscript to be freely available to other researchers, either 1. In a public repository, 2. Within the manuscript itself, or 3. Uploaded as supplementary information.

**Response:** Data used in this paper have been included within the manuscript itself including references where the data are published.

3. Figure 1 appears to have been adapted from a previously published figure. Please provide written permission from the copyright holder to publish this under our CC-BY 4.0 license, or remove the figure / replace the image. Please note we do not recommend using standard request forms available on Publishers' websites, as they grant single use rather than republication under an open access license

**Response:** Figure 1 is an original image that was created using published data. We have modified the legend in Figure 1 to clarify: “***Map created by Cruz Dragonasvac Graphics using published data from Stellman al. 2003 and Vietnam Soils and Fertilizer Institute, 1999.”***

**Authors’ Response to Reviewers’ Comments**

**Reviewer #1:** The manuscript is informative and merits publication to bring about awareness for prevention of CHD caused by conscious and unconscious presence of dioxin TCDD residuals from the use of 2, 4, 5-T herbicides (Agent Orange) in the soil. However it is expected that the author should provide full forms of Types of CHD in Table 1. Distribution of CHD sub-types in the Da Nang Region, 2015-201

**Response:** While the Liu et al. CHD comparison paper had 27 CHD subtypes – our table only contains sub-types that were found in the DaNang Region between 2015 and 2016. All the other sub-types were zeros thus we did not include them in the table.

**Reviewer #2, comment 1:** The study is important in the context of conflict-prone region. However, the manuscript should be improved substantially before accepting for publication. Please follow the journal instruction to write the Method section. The methodological description are not comprehensive. Line 148: authors repeatedly mentioned that in the "original study" it is not clear how this study is different the previous one. I would suggest to include if authors tested statistical difference in the sub-group analysis. Please present effect estimates in two digits prior to and after decimal point.

**Response**: The authors have edited the paper by 1) elaborating the Methods section by further describing the data evaluation and categorization process to distinguish the methods between the original study and the current study, and 2) clarified our language in referencing the difference between the previous studies results and our current study results to be more clear and succinct. We use the term “original” to represent the 2019 Giang et al data; and the term “current” to denote our reanalysis of the 2019 Giang et al paper that are presented in this 2022 paper. Also, we changed all our birth prevalence rates into 2 digits after the decimal point in our revised manuscript.

**Reviewer #2, comment 2**: The manuscript is poorly written or interpreted the results. Authors were more interested in highlighting the p-values rather than interpretating the results. The comparison authors made with previous study should be clear and concise because readability is low. For example in line 179-186: "This resulted in a revised Da Nang region overall CHD birth prevalence rate 14.712/1000 live births 177 (95% CI 12.747-16.690) and when eliminating singular PDAs, which are a common finding at birth, the Da Nang region CHD birth prevalence rate dropped to 12.207/1000 live births (95% CI 10.470-14.005). These data were then compared to CHD prevalence data for Asia published by Liu et al. of 9.342/1000 (95% CI 8.072-10.704). Statistical analysis of these two CHD birth prevalence rates resulted in a significantly higher overall Da Nang CHD birth prevalence rate (p<0.0001) and when singular PDA were eliminated (p<0.0001)."

Findings will be more readable if authors could revise it by a language expert. I apologize if it sounds rude.

Discussion should be precise because most of the information are repeated.

**Response:** The above paragraph was revised. Also, the whole manuscript was edited to be more clear and succinct.

Please let us know if you require any further information.

Sincerely,

Hoang Thi Nam Giang, MBBS

Tran Thanh Hai, MD

Hoang Nguyen, MD

Thanh K. Vuong, MD

Lois Wright Morton, PhD

Casey B. Culbertson, MD
